# Time Trends in the Diagnosis of Colorectal Cancer With Obstruction, Perforation, and Emergency Admission After the Introduction of Population-Based Organized Screening

**DOI:** 10.1001/jamanetworkopen.2020.5741

**Published:** 2020-05-26

**Authors:** Kathleen M. Decker, Pascal Lambert, Zoann Nugent, Natalie Biswanger, Jewel Samadder, Harminder Singh

**Affiliations:** 1 Department of Community Health Sciences, University of Manitoba, Winnipeg, Manitoba; 2 Research Institute in Oncology and Hematology, CancerCare Manitoba, Winnipeg, Manitoba; 3 Epidemiology and Cancer Registry, CancerCare Manitoba, Winnipeg, Manitoba; 4 Department of Internal Medicine, University of Manitoba, Winnipeg, Manitoba; 5 Division of Gastroenterology and Hepatology, Department of Medicine, Mayo Clinic, Phoenix, Arizona; 6 Department of Medicine, University of Utah, Salt Lake City; 7 Medical Oncology and Hematology, CancerCare Manitoba, Winnipeg, Manitoba

## Abstract

**Importance:**

Up to 30% of patients with a diagnosis of colorectal cancer (CRC) present as an emergency (an intestinal obstruction, perforation, or emergency hospital admission) (OPE). There are limited data about the association of organized, population-based colorectal cancer screening with the rate of emergency presentations.

**Objective:**

To examine the association of CRC screening with OPE at cancer diagnosis and time trends in the rate of OPE after the start of organized CRC screening using a highly sensitive fecal occult blood test.

**Design, Setting, and Participants:**

A historical cohort study was conducted among 1861 individuals 52 to 74 years of age with a diagnosis of CRC from January 1, 2007, to December 31, 2015, who lived in Winnipeg, Manitoba, a province with universal health care and an organized CRC screening program. Statistical analysis was performed from January 22, 2019, to February 26, 2020.

**Exposures:**

Variables included prior CRC screening, era of diagnosis, cancer stage at diagnosis, tumor site in the colon, area level mean household income, primary care continuity of care, and comorbidity.

**Main Outcomes and Measures:**

The primary outcomes were defined as an OPE. Logistic regression was used to evaluate factors associated with OPE at CRC diagnosis. Trends over time were calculated using Joinpoint Regression.

**Results:**

From 2007 to 2015, 1861 individuals 52 to 74 years of age (1133 men; median age, 65.1 years [interquartile range, 60.0-70.3 years]) received a diagnosis of CRC in Winnipeg. Most individuals had good continuity of care and moderate comorbidities. Overall, 345 individuals (18.5%) had an OPE. The rate of emergency hospital admissions decreased significantly from 2007 (the start of the organized, province-wide CRC screening program) to 2015 (annual change, –7.1%; 95% CI, –11.3% to –2.8%; *P* = .01). There was no change in the rate of obstructions or perforations or stage IV CRCs. Individuals who were up to date for CRC screening were significantly less likely to receive a diagnosis of an OPE (odds ratio, 0.38; 95% CI, 0.28-0.50; *P* < .001). The results were similar after adding emergency department visits and stage IV CRC at diagnosis to the outcome.

**Conclusions and Relevance:**

This study suggests that the rate of emergency hospital admissions decreased over time for individuals who underwent CRC screening, but there was no change in the rate of obstructions and perforations. Individuals who were up to date for CRC screening were less likely to have a CRC diagnosis with an OPE.

## Key Points

**Question:**

What is the association of organized, population-based colorectal cancer screening with the rate of obstructions, perforations, and emergency admissions prior to a colorectal cancer diagnosis?

**Findings:**

This cohort study of 1861 Canadian adults with a diagnosis of colorectal cancer found no change in the rate of obstructions and perforations, but found a significant decrease in the rate of emergency hospital admissions after the implementation of organized colorectal cancer screening. Individuals who were up to date for colorectal screening were less likely to receive a diagnosis of an obstruction or perforation or have an emergency admission.

**Meaning:**

Targeted colorectal cancer screening strategies are needed that focus on individuals at higher risk of an emergency presentation.

## Teaser

This cohort study examines the association of colorectal cancer screening with an intestinal obstruction, perforation, or emergency hospital admission at diagnosis, as well as trends in the rate of these outcomes after the start of colorectal cancer screening using a highly sensitive fecal occult blood test.

## Introduction

Colorectal cancer (CRC) is the second most common cancer in North America, accounting for 13% of all cancer diagnoses in Canada.^1^ Although the incidence of CRC has decreased during the last 20 years, one-half of individuals with CRC receive the diagnosis when the cancer is in a late stage.^1,2^ In addition, 15% to 30% of patients with a CRC diagnosis present as an emergency.^3,4,5^ Emergencies include intestinal obstructions, perforations, or emergency admissions to the hospital (OPE) prior to diagnosis.^6^ Short-term^7,8^ and long-term^9^ survival for individuals with a diagnosis of CRC after an OPE is worse compared with nonemergency diagnoses even when adjusted for age and comorbidity.^10^ Moreover, the health care burden of emergency CRC presentations is substantial, as these patients spend greater than 50% more days in the hospital than those with nonemergency diagnoses and overall treatment costs are higher.^11^ Therefore, the rate of OPE among individuals who present with a new CRC diagnosis is a useful quality indicator because it represents a missed opportunity to diagnose CRC early.^12,13,14,15,16^

One strategy that may decrease OPE rates is screening.^17^ In 1998, Scholefield et al^18^ examined the association of screening with emergency CRC presentations (N = 150 251) using the fecal occult blood test (FOBT). There were significantly fewer emergencies in the group with CRC detected via FOBT compared with the control group. An evaluation of a fast-track flexible sigmoidoscopy (FS) service in the UK (N = 2294) also found a nonstatistically significant reduction in emergency CRC presentations.^19^ However, data are limited on the association between emergency CRC diagnoses in the population and the implementation of organized, population-based CRC screening.

The Canadian Task Force on Preventive Health Care recommends screening for individuals 50 to 74 years of age with an FOBT or fecal immunochemical test every 2 years or FS every 10 years.^20^ All Canadian provinces have implemented or are in the process of implementing organized CRC screening programs.^1^ Manitoba initiated a population-based CRC screening program (ColonCheck) in 2007 administered by CancerCare Manitoba. ColonCheck mails the high-sensitive FOBT Hemoccult Sensa (HemoCue) every 2 years to eligible Manitobans 50 to 74 years of age. Residents may also complete an FOBT provided by their primary care clinician (PCC; nonspecialty clinicians who provide general, day-to-day health care). Rates of FOBTs have increased since the start of the program, from 32.2% in 2008 to 41.6% in 2012.^21,22^ However, the association of population-based CRC screening with delays to diagnosis such as OPE have not been evaluated, to our knowledge.

In this study, we used administrative health data to examine the association of CRC screening with OPE among individuals with a diagnosis of CRC. We examined time trends in the rate of OPE, emergency department (ED) visits, and diagnoses of stage IV CRC after the start of organized CRC screening, as well as factors associated with OPE including up-to-date CRC screening status for patients with CRC at initial presentation in a population-based setting.

## Methods

### Data Sources

The Manitoba Cancer Registry was used to identify individuals with a diagnosis of CRC, as well as their sex, diagnosis date, tumor location, and death date. The Manitoba Cancer Registry is a population-based registry that is legally mandated to collect, classify, and maintain accurate, comprehensive information about cancer cases in the province of Manitoba, Canada. The Manitoba Cancer Registry has been shown to be of very high quality, including high levels of completeness and histologic verification.^23^ This study was approved by the University of Manitoba’s Health Research Ethics Board, Manitoba Health’s Health Information and Privacy Committee, and CancerCare Manitoba’s Research and Resource Impact Committee. Because data were deidentified, informed consent was not required. This study followed the Strengthening the Reporting of Observational Studies in Epidemiology (STROBE) reporting guideline.

Manitoba Health, the publicly funded provincial health insurance agency, provides comprehensive universal health coverage for hospitalizations, procedures, and physician visits for provincial residents (approximately 1.35 million in 2018). Manitoba Health maintains several electronic databases to monitor health care use and reimburse clinicians for services delivered. Since 1984, provincial residents have been assigned a personal health identification number that can be used to link provincial health information databases, allowing health care use and outcomes to be tracked longitudinally.

We used 3 Manitoba Health administrative databases: the Manitoba Population Registry, the Medical Claims database, and the Hospital Abstracts database. The Manitoba Population Registry contains demographic, vital status, and migration information and was used to assess the duration of provincial health coverage. The Medical Claims database is generated by claims filed by health care professionals for reimbursement of service. Medical Claims data were used to identify outpatient contacts with the health care system and type of contact (PCC or specialist), colonoscopy, FS, and nonprogram FOBT use. The Hospital Abstracts database includes all hospital admissions for Manitoba residents and was used to identify emergency and nonemergency hospital admissions regardless of admission route. Medical Claims and Hospital Abstracts data were also used to examine comorbidities and identify individuals with a diagnosis of ulcerative colitis or Crohn disease using a previously validated algorithm.^24^ The accuracy and completeness of Manitoba Health’s administrative data has been previously established.^25,26,27^

The Winnipeg Regional Health Authority administers the delivery of health care in Winnipeg (two-thirds of the provincial population). Winnipeg ED visits that did not lead to hospitalization were identified using the Winnipeg Regional Health Authority’s Admissions, Discharge and Transfer and E-Triage data and Emergency Department Information System databases. ColonCheck’s population-based registry was used to identify individuals who completed a screening program FOBT. Statistics Canada 2006 census data were used to evaluate the area-level mean household income based on each individual’s area of residence.^28,29,30^

### Study Design and Population

This study used a population-based historical cohort design. Individuals 52 to 74 years of age with a diagnosis of CRC (*International Classification of Diseases, Oncology, Third Revision* [*ICD-O-3*] codes C18.0. C18.2-9, C19, C20, and C26.0) from January 1, 2007, to December 31, 2015, who lived in Winnipeg at diagnosis were included. Individuals who lived outside of Winnipeg were excluded because FOBTs in rural and northern areas of Manitoba are not registered in the Medical Claims database and only Winnipeg EDs are included in the ED database. Individuals 52 years of age were included because ColonCheck recommends that screening begin at 50 years of age and the study required at least 2 years of screening information to determine screening history. Only individuals with a first diagnosis of CRC were included. Individuals with a prior diagnosis of ulcerative colitis or Crohn disease were excluded, because these individuals are at higher than average risk of CRC and are closely followed up by health care professionals.

### Definition of Variables

Up-to-date screening was defined as a program or nonprogram FOBT in the previous 2 years, FS in the previous 5 years (recommended interval during most of the study years), or colonoscopy in the previous 10 years. Program FOBTs included FOBTs provided by ColonCheck. Nonprogram FOBTs included FOBTs provided outside of the screening program (ie, by PCC or health clinics). Flexible sigmoidoscopy and colonoscopies in the 3 months prior to CRC diagnosis were excluded, as they were likely to be for diagnostic purposes.^21^ Program and nonprogram FOBTs that occurred up to 30 days before diagnosis were also excluded, as the primary outcome assessed was OPE in the month prior to CRC diagnosis. Colonoscopies, FS, and program and nonprogram FOBTs were included as separate variables in the univariable models. Era of diagnosis was separated into 2007 to 2010 (early program screening) and 2011 to 2015 (established program screening). Cancer stage at diagnosis was classified using the American Joint Committee on Cancer collaborative staging system.^31^ Colorectal tumor site was classified as cecum (*ICD-O-3* code C18.0), ascending colon (*ICD-O-3* code C18.2), hepatic flexure (*ICD-O-3* code C18.3), transverse colon (*ICD-O-3* code C18.4), splenic flexure (*ICD-O-3* code C18.5), descending colon (*ICD-O-3* code C18.6), sigmoid colon (*ICD-O-3* code C18.7), overlapping lesion of the colon (*ICD-O-3* code C18.8) rectosigmoid junction (*ICD-O-3* code C19.9), rectum (*ICD-O-3* code C20.9), and colon, not otherwise specified or unknown (*ICD-O-3* code C18.9). Colorectal cancer tumor site was compressed into 5 categories for the logistic regression: cecum, ascending colon, and hepatic flexure; transverse colon and splenic flexure; descending colon and sigmoid colon; rectosigmoid junction; and rectum.

Area-level mean household income was categorized by quintile from lowest (income quintile 1) to highest (income quintile 5). Primary care clinician continuity of care (whether or not an individual received most of their ambulatory care from a single nonspecialist clinician) was measured by identifying individuals with at least 50% of visits to the same PCC among those with at least 3 visits in the 6 to 30 months prior to diagnosis.^32^ Comorbidity was measured by the resource use band calculated using the Johns Hopkins Adjusted Clinical Group System software.^33^ The resource use band includes the following 6 categories: 0 indicates nonuser; 1, healthy user; 2, low morbidity; 3, moderate morbidity; 4, high morbidity; and 5, very high morbidity. The resource use band is based on age, sex, physician claims, and hospital discharges in the year prior to diagnosis.

### Outcomes

The primary outcomes were intestinal obstruction, intestinal perforation, or emergency hospital admission. Intestinal obstructions and perforations were identified using *International Statistical Classification of Diseases and Related Health Problems, Tenth Revision* (*ICD-10*) code K56.6 (other and unspecified intestinal obstruction) and *ICD-10* code K63.1 (perforation of the intestine, nontraumatic).^13^ An emergency hospital admission was identified using the emergency entry code (ie, patients who have a life-threatening condition requiring immediate assessment, treatment, and admission to the hospital).^34^ This includes all modes of entry to the hospital and does not necessarily mean that the patient entered the hospital via the ED. Emergency department visits in the 30 days prior to a CRC diagnosis and diagnosis of stage IV CRC were added as outcomes in sensitivity analyses.

### Statistical Analysis

Statistical analysis was performed from January 22, 2019, to February 26, 2020. Univariable and multivariable logistic regression analyses were used to evaluate factors associated with OPE. The following factors were included: screening history (up to date, colonoscopy, FS, program FOBT, and nonprogram FOBT), sex, age, income quintile, CRC stage at diagnosis, tumor location, continuity of care, comorbidity, era of diagnosis, PCC visits, specialist visits, and hospitalizations.^13^ For CRC screening history, only up-to-date screening was included in the multivariable model.

Descriptive statistics and logistic regression analyses were performed with SAS, version 9.4 (SAS Institute Inc). Trends over time (rate per 1000 individuals), annual percentage change (APC),^35^ and 95% CIs were calculated using the Joinpoint Regression program, version 4.2.0.1, developed by the Surveillance Epidemiology and End Results program (National Cancer Institute) using a Poisson regression model.

## Results

From 2007 to 2015, 1861 individuals 52 to 74 years of age received a diagnosis of CRC in Winnipeg. Most were male (1133 [60.9%]), had good continuity of care (1438 [77.3%]), moderate comorbidities (resource use band, 3; 1092 [58.7%]), and 433 (23.3%) were up to date for screening (Table 1). Overall, 345 (18.5%) had an OPE and 202 (10.9%) had an ED visit in the month prior to diagnosis. Fewer individuals with OPE than those without OPE had a program FOBT (14 of 345 [4.1%] vs 144 of 1516 [9.5%]) or nonprogram FOBT (87 of 345 [25.2%] vs 719 of 1516 [47.4%]), FS (<6 of 345 [exact data censored because the count is <6] vs 24 of 1516 [1.6%]), or colonoscopy (37 of 345 [10.7%] vs 245 of 1516 [16.2%]) and were less likely to be up to date for screening (50 of 345 [14.5%] vs 383 of 1516 [25.3%]). Individuals with OPE tended to be older (median age, 66 years [interquartile range, 60-70 years] vs 64 years [interquartile range, 59-70 years]), female (152 of 345 [44.1%] vs 576 of 1516 [38.0%]), with a diagnosis of a later stage cancer (stage IV, 142 of 345 [41.2%] vs 273 of 1516 [18.0%]), have lower income (lowest quintile, 89 of 345 [25.8%] vs 275 of 1516 [18.1%]), higher comorbidity (resource use band, 5: 14 of 345 [4.1%] vs 40 of 1516 [2.6%]), and have fewer prior specialist visits (91-181 days: mean [SD], 0.4 [1.0] vs 0.5 [1.2]). Continuity of care was similar between patients with CRC who had or did not have OPE, although more individuals with OPE had fewer than 3 visits to a PCC in the year before diagnosis. The most frequently diagnosed colonic locations of CRC for individuals with OPE were the sigmoid colon (73 of 345 [21.2%]), cecum (67 of 345 [19.4%]), or rectum (71 of 345 [20.6%]). The most frequently diagnosed location for individuals without an OPE was the rectum (508 of 1516 [33.5%]).

**Table 1.** Characteristics of Individuals 52 to 74 Years of Age With a Diagnosis of Colorectal Cancer With and Without OPE, 2007-2015, Winnipeg, Manitoba

Characteristic	OPE, No. (%)		
	No (n = 1516)	Yes (n = 345)	*P* value
Age, median (IQR), y	64.9 (59.8-70.3)	66.1 (60.8-70.6)	.03
Screening status			
Up to date^a^	383 (25.3)	50 (14.5)	<.001
Not up to date	1133 (74.7)	295 (85.5)	
Program FOBT			
Yes	144 (9.5)	14 (4.1)	.001
No	1372 (90.5)	331 (95.9)	
Nonprogram FOBT			
Yes	719 (47.4)	87 (25.2)	<.001
No	797 (52.6)	258 (74.8)	
Flexible sigmoidoscopy			
Yes	24 (1.6)	Suppressed^b^	.45
No	1492 (98.4)	Suppressed^b^	
Colonoscopy			
Yes	245 (16.2)	37 (10.7)	.01
No	1271 (83.8)	308 (89.3)	
Era of diagnosis			
2007-2010	657 (43.3)	183 (53.0)	.001
2011-2015	859 (56.7)	162 (47.0)	
Sex			
Male	940 (62.0)	193 (55.9)	.04
Female	576 (38.0)	152 (44.1)	
Tumor location			
Cecum	210 (13.9)	67 (19.4)	<.001
Ascending colon	148 (9.8)	47 (13.6)	
Hepatic flexure	46 (3.0)	7 (2.0)	
Transverse colon	79 (5.2)	25 (7.3)	
Splenic flexure	37 (2.4)	13 (3.8)	
Descending colon	41 (2.7)	6 (1.7)	
Sigmoid colon	306 (20.2)	73 (21.2)	
Overlapping lesion of colon and unspecified	13 (0.9)	11 (3.2)	
Rectosigmoid junction	128 (8.4)	25 (7.3)	
Rectum	508 (33.5)	71 (20.6)	
Stage			
I	338 (22.3)	17 (4.9)	<.001
II	369 (24.3)	93 (27.0)	
III	536 (35.4)	93 (27.0)	
IV	273 (18.0)	142 (41.2)	
Income quintile			
1 (lowest)	275 (18.1)	89 (25.8)	<.001
2	282 (18.6)	82 (23.8)	
3	285 (18.8)	57 (16.5)	
4	338 (22.3)	60 (17.4)	
5 (highest)	317 (20.9)	47 (13.6)	
Unknown	19 (1.2)	10 (2.9)	
Continuity of care^c^			
≥50% (Yes)	1188 (78.4)	250 (72.5)	.02
<50% (No)	130 (8.6)	31 (9.0)	
<3 Visits	198 (13.1)	64 (18.6)	
Comorbidities (resource use band)			
0 (Nonuser)	130 (8.6)	59 (17.1)	<.001
1 (Healthy user)	52 (3.4)	10 (2.9)	
2 (Low morbidity)	254 (16.8)	42 (12.2)	
3 (Moderate morbidity)	900 (59.4)	192 (55.7)	
4 (High morbidity)	140 (9.2)	28 (8.1)	
5 (Very high morbidity)	40 (2.6)	14 (4.1)	
**Primary care clinician visits**			
From 91 to 181 d			
Mean (SD)	1.7 (1.9)	1.8 (2.1)	
Median (IQR)	1 (0-2)	1 (0-3)	.63
≥1 Visit	1076 (71.0)	223 (64.6)	.02
From 182 to 272 d			
Mean (SD)	1.4 (1.7)	1.6 (2.1)	
Median (IQR)	1 (0-2)	1 (0-2)	.89
≥1 Visit	981 (64.7)	214 (62.0)	.35
From 273 to 365 d			
Mean (SD)	1.4 (1.6)	1.6 (2.1)	
Median (IQR)	1 (0-2)	1 (0-2)	.44
≥1 Visit	955 (63.0)	210 (60.9)	.46
**Specialist visits**			
From 91 to 181 d			
Mean (SD)	0.5 (1.2)	0.4 (1.0)	
Median (IQR)	0 (0-1)	0 (0-0)	.25
≥1 Visits	394 (26.0)	78 (22.6)	.22
From 182 to 272 d			
Mean (SD)	0.4 (1.3)	0.3 (1.0)	
Median (IQR)	0 (0-0)	0 (0-0)	.96
≥1 Visits	279 (18.4)	63 (18.3)	>.99
From 273 to 365 d			
Mean (SD)	0.3 (1.1)	0.4 (1.2)	
Median (IQR)	0 (0-0)	0 (0-0)	.58
≥1 Visits	251 (16.6)	61 (17.7)	.64
**Emergency department visits**			
From 31 to 90 d			
Mean (SD)	0.1 (0.5)	0.5 (1.3)	
Median (IQR)	0 (0-0)	0 (0-0)	.001
≥1 Visits	133 (8.8)	49 (14.2)	.003
From 91 to 181 d			
Mean (SD)	0.1 (0.4)	0.2 (0.6)	
Median (IQR)	0 (0-0)	0 (0-0)	.01
≥1 Visits	113 (7.5)	41 (11.9)	.01
From 182 to 272 d			
Mean (SD)	0.1 (0.3)	0.1 (0.3)	
Median (IQR)	0 (0-0)	0 (0-0)	.02
≥1 Visits	84 (5.5)	31 (9.0)	.02
From 273 to 365 d			
Mean (SD)	0.1 (0.3)	0.1 (0.4)	
Median (IQR)	0 (0-0)	0 (0-0)	.01
≥1 Visits	65 (4.3)	27 (7.8)	.01
**Hospital admissions**			
From 31 to 90 d			
Mean (SD)	0.03 (0.2)	0.05 (0.2)	
Median (IQR)	0 (0-0)	0 (0-0)	.04
≥1 Visits	42 (2.8)	17 (4.9)	.06
From 91 to 181 d			
Mean (SD)	0.03 (0.2)	0.1 (0.3)	
Median (IQR)	0 (0-0)	0 (0-0)	.03
≥1 Visits	41 (2.7)	17 (4.9)	.04
From 182 to 272 d			
Mean (SD)	0.02 (0.1)	0.03 (0.2)	
Median (IQR)	0 (0-0)	0 (0-0)	.13
≥1 Visits	22 (1.5)	9 (2.6)	.16
From 273 to 365 d			
Mean (SD)	0.02 (0.2)	0.02 (0.2)	
Median (IQR)	0 (0-0)	0 (0-0)	.90
≥1 Visits	29 (1.9)	7 (2.0)	.83

Abbreviations: FOBT, fecal occult blood test; OPE, obstructions, perforations, or emergency admissions; IQR, interquartile range.

^a^ Up-to-date screening is defined as a program or nonprogram FOBT in the previous 2 years, flexible sigmoidoscopy in the previous 5 years, or a colonoscopy in the previous 10 years (excluding nonprogram FOBTs, flexible sigmoidoscopies, and colonoscopies in the 3 months prior to diagnosis).

^b^ Owing to counts of less than 6.

^c^ Continuity of care includes primary care clinician visits in the 6-30 months prior to diagnosis.

The OPE rate significantly decreased from 2007 to 2015 (APC, –6.2; 95% CI, –10.0 to –2.2; *P* = .01) (Figure), primarily due to the significant decrease in the emergency hospital admission rate (APC, –7.1; 95% CI, –11.3 to –2.8; *P* = .01). There was no significant change over time in the rate of obstructions and perforations (APC, –1.8; 95% CI, –15.0 to 13.5; *P* = .78), ED visits (APC, –0.8; 95% CI, –3.9 to 2.4; *P* = .57), or diagnoses of stage IV CRC (APC, 0.1; 95% CI, –4.6 to 5.1; *P* = .95).

**Figure.**
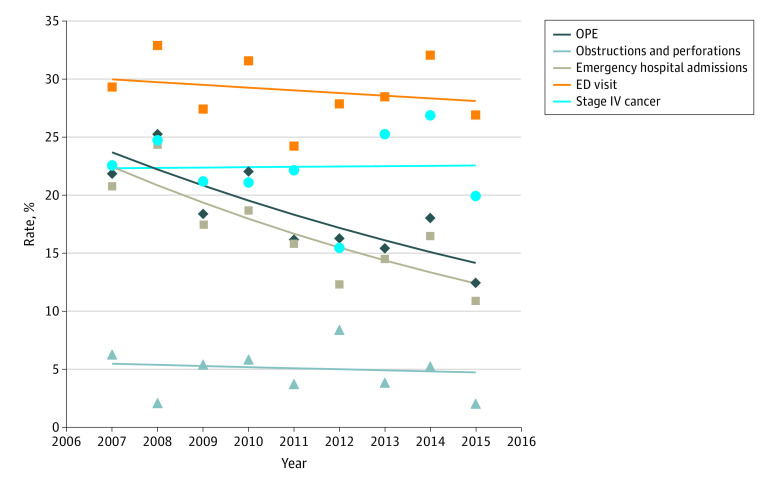
Rate of Obstruction, Perforation, Emergency Hospital Admissions (OPE), and Diagnoses of Stage IV Colorectal Cancer Over Time Change in the rate of OPE, obstructions and perforations, emergency hospital admissions, emergency department (ED) visits, and diagnoses of stage IV cancer for 1861 individuals with a diagnosis of colorectal cancer, 2007-2015 (annual rate represented by symbols with trend line added).

In the univariable logistic regression model, individuals who had a program FOBT (odds ratio [OR], 0.40; 95% CI, 0.23-0.71) or nonprogram FOBT (OR, 0.37; 95% CI, 0.29-0.49), had a colonoscopy (OR, 0.62; 95% CI, 0.43-0.90), or were up to date for screening (OR, 0.32; 95% CI, 0.25-0.41) were significantly less likely to have OPE (Table 2). In the multivariable model that included all variables and up-to-date screening (a composite measure of screening that includes FOBT, FS, and colonoscopy), individuals who were older (OR, 1.03; 95% CI, 1.01-1.06), lived in a lower-income area (lowest quintile: OR, 1.69; 95% CI, 1.11-2.59), had a tumor not located in the rectum (cecum, ascending colon, or hepatic flexure: OR, 2.61; 95% CI, 1.82-3.74; transverse colon or splenic flexure: OR, 3.13; 95% CI, 1.92-5.12; and descending colon or sigmoid colon: OR, 1.79; 95% CI, 1.21-2.63), no comorbidity (OR, 2.12; 95% CI, 0.92-4.89), or were diagnosed in 2007 to 2010 (OR, 1.39; 95% CI, 1.07-1.81) were significantly more likely to have OPE, while those with a stage I or II CRC (OR, 0.35; 95% CI, 0.25-0.49), a PCC visit 31 to 90 days prior to diagnosis (OR, 0.69; 95% CI, 0.51-0.93) or specialist visit 31 to 90 days prior to diagnosis (OR, 0.56; 95% CI, 0.40-0.78) were less likely to have OPE. Individuals who were up to date for screening were significantly less likely to experience an OPE (OR, 0.38; 95% CI, 0.28-0.50; *P* < .001). The results were similar after adding ED visits and stage IV CRC at diagnosis to the outcome (eTables 1 and 2 in the Supplement).

**Table 2.** Factors Associated With OPE for Individuals With a Diagnosis of Colorectal Cancer, 52 to 74 Years of Age, Winnipeg, 2007-2015

Variable	Univariable		Multivariable	
	OR (95% CI)	*P* value^a^	OR (95% CI)	*P* value
Sex				
Female	1.29 (1.02-1.63)	.04	1.21 (0.92-1.58)	.17
Male	1 [Reference]		1 [Reference]	
Age	1.02 (1.00-1.04)	.02	1.03 (1.01-1.06)	.003
Income quintile				
1 (Lowest)	2.18 (1.48-3.22)	<.001	1.69 (1.11-2.59)	.006
2	1.96 (1.32-2.91)		1.66 (1.08-2.54)	
3	1.35 (0.89-2.05)		1.09 (0.69-1.73)	
4	1.20 (0.79-1.81)		0.95 (0.61-1.49)	
5 (Highest)	1 [Reference]		1 [Reference]	
Stage				
I or II	0.30 (0.23-0.4)	<.001	0.35 (0.25-0.49)	<.001
III	0.33 (0.25-0.45)		0.37 (0.27-0.52)	
IV	1 [Reference]		1 [Reference]	
Tumor location				
Cecum, ascending colon, or hepatic flexure	2.14 (1.56-2.95)	<.001	2.61 (1.82-3.74)	<.001
Transverse colon or splenic flexure	2.34 (1.51-3.65)		3.13 (1.92-5.12)	
Descending colon or sigmoid colon	1.63 (1.15-2.31)		1.79 (1.21-2.63)	
Rectosigmoid junction	1.40 (0.85-2.29)		1.36 (0.79-2.33)	
Rectum	1 [Reference]		1 [Reference]	
Continuity of care^b^				
≥50%	0.88 (0.58-1.34)	.03	1.00 (0.62-1.60)	.80
<3 Visits	1.36 (0.84-2.2)		0.82 (0.40-1.65)	
<50%	1 [Reference]		1 [Reference]	
Screening history				
Up to date^c^	0.32 (0.25-0.41)	<.001	0.38 (0.28-0.50)	<.001
Not up to date	1 [Reference]		1 [Reference]	
Program FOBT	0.40 (0.23-0.71)	.001	NA	NA
No program FOBT	1 [Reference]		NA	
Colonoscopy	0.62 (0.43-0.90)	.01	NA	NA
No colonoscopy	1 [Reference]		NA	
Flexible sigmoidoscopy	0.55 (0.16-1.82)	.32	NA	NA
No flexible sigmoidoscopy	1 [Reference]		NA	
Nonprogram FOBT	0.37 (0.29-0.49)	<.001	NA	NA
No nonprogram FOBT	1 [Reference]		NA	
Comorbidities (resource use band)				
0 (Nonuser)	2.26 (1.12-4.96)	.11	2.12 (0.92-4.89)	.05
1 (Healthy user)	1 [Reference]		1 [Reference]	
2 (Low morbidity)	0.86 (0.41-1.82)		0.82 (0.35-1.90)	
3, 4, 5 (3, Moderate morbidity; 4, high morbidity; and 5, very high morbidity)	1.13 (0.56-2.25)		0.91 (0.40-2.07)	
Era				
2007-2010	1.48 (1.17-1.87)	.001	1.39 (1.07-1.81)	.02
2011-2015	1 [Reference]		1 [Reference]	
Primary care clinician visits				
From 31 to 90 d				
≥1	0.65 (0.51-0.83)	.001	0.69 (0.51-0.93)	.01
0	1 [Reference]		1 [Reference]	
From 91 to 181 d				
≥1	0.75 (0.58-0.96)	.02	0.92 (0.67-1.26)	.60
0	1 [Reference]		1 [Reference]	
From 182 to 272 d				
≥1	0.89 (0.7-1.13)	.35	1.17 (0.82-1.65)	.40
0	1 [Reference]		1 [Reference]	
From 273 to 365 d				
≥1	0.91 (0.72-1.16)	.46	1.20 (0.86-1.69)	.29
0	1 [Reference]		1 [Reference]	
Specialist visits				
From 31 to 90 d				
≥1	0.54 (0.41-0.71)	<.001	0.56 (0.40-0.78)	.001
0	1 [Reference]		1 [Reference]	
From 91 to 181 d				
≥1	0.85 (0.64-1.12)	.24	0.91 (0.63-1.31)	.62
0	1 [Reference]		1 [Reference]	
From 182 to 272 d				
≥1	0.98 (0.72-1.34)	.91	1.02 (0.68-1.52)	.93
0	1 [Reference]		1 [Reference]	
From 273 to 365 d				
≥1	1.05 (0.77-1.45)	.76	1.38 (0.91-2.08)	.13
0	1 [Reference]		1 [Reference]	
Hospital admissions				
From 31 to 90 d				
≥1	1.82 (1.02-3.24)	.04	1.77 (0.87-3.59)	.11
0	1 [Reference]		1 [Reference]	
From 91 to 181 d				
≥1	1.87 (1.05-3.32)	.03	1.67 (0.83-3.36)	.15
0	1 [Reference]		1 [Reference]	
From 182 to 272 d				
≥1	1.82 (0.83-3.99)	.13	1.87 (0.74-4.71)	.19
0	1 [Reference]		1 [Reference]	
From 273 to 365 d				
≥1	1.06 (0.46-2.45)	.88	0.61 (0.21-1.74)	.35
0	1 [Reference]		1 [Reference]	

Abbreviations: FOBT, fecal occult blood test; NA, not applicable; OPE, obstructions, perforations, or emergency admissions; OR, odds ratio.

^a^ Type 3 *P* value reported.

^b^ Continuity of care includes primary care clinician visits in the 6 to 30 months prior to diagnosis.

^c^ Up-to-date screening is defined as a program or nonprogram FOBT in the previous 2 years, flexible sigmoidoscopy in the previous 5 years, or a colonoscopy in the previous 10 years (excluding nonprogram FOBTs, flexible sigmoidoscopies, and colonoscopies in the 3 months prior to diagnosis).

## Discussion

We found that the overall OPE rate decreased significantly from 2007, when provincial organized CRC screening was implemented, to 2015. This trend was primarily owing to a decrease in the rate of emergency hospital admissions. There was no change in the rate of obstructions and perforations or stage IV CRCs. A history of up-to-date CRC screening was independently associated with a decreased risk of CRC presentation with OPE.

A study from Ontario (N = 59 670) found a 31% decrease in the percentage of individuals with CRC who presented with OPE during 1999-2001 compared with 1993-1995.^14^ That study did not examine obstructions and perforations separately from emergency hospital admissions and was conducted in the prescreening program era.^36^ Hwang^37^ compared emergency and nonemergency presentations of CRC at 1 hospital in British Columbia during 2009-2010 (N = 75). Overall, 43% of patients presented on an emergency basis. That study was also conducted prior to the implementation of organized CRC screening. In the United Kingdom (UK), Pande et al^38^ compared the number of patients with CRC presenting as an emergency prior to the start of a CRC awareness campaign at 1 hospital during a 6-month period. The campaign was associated with a 62.5% decrease in the number of patients with CRC presenting as an emergency (N = 26). In 2017, Askari et al^10^ found a significant decrease in CRC emergency presentations in the UK among individuals 60 to 69 years of age (N = 286 591), from 23.4% before organized screening (1997-2005) to 14.9% after screening (2006-2012) (*P* < .001).

The lack of decline in the rate of obstructions and perforations or stage IV CRCs after the introduction of organized CRC screening in Winnipeg is interesting. Previous research found that the CRC detection sensitivity for the Hemoccult Sensa FOBT used in Manitoba was 63.2%.^39^ This sensitivity is at the lower end of the range reported from other studies and similar to that reported for older guaiac-based FOBTs.^40,41^ The low sensitivity of of the Hemoccult Sensa FOBT, combined with the fact that 40% to 50% of individuals 50 to 74 years of age are not up to date for CRC screening,^22^ could be associated with the lack of decline observed in obstructions and perforations. However, we found that up-to-date CRC screening was associated with a significantly lower risk of OPE, which supports the importance of CRC screening in reducing OPE. More important, the rate of up-to-date screening for individuals with CRC in our study is lower than that in the overall population, suggesting that those at most risk of CRC are the least likely to undergo screening. Whether the use of alternative CRC screening tests (eg, fecal immunochemical test, which has a higher sensitivity in observational studies and increased acceptability compared with guaiac-based FOBTs)^42,43^ would lead to higher CRC screening uptake among those who need it the most in North America needs to be determined. The effect of longer follow-up after the onset of population-based screening will also need to be evaluated in future studies.

Our study found that individuals with CRC who had lower mean household incomes were more likely to have OPE. These results are consistent with other studies that have found socioeconomic differences among patients with a diagnosis of CRC through emergency routes despite the provision of universal health care.^12,44,45^ The association between income and the increased risk of OPE may be owing to individuals seeking health care only after developing advanced symptoms and lower screening rates.^21^ We also found that, while individuals who had recently visited a PCC or specialist were less likely to present with OPE, many individuals with OPE had good continuity of care and had visited a PCC or specialist in the months prior to a CRC diagnosis. This finding suggests that there may be missed opportunities to prevent OPE. In an examination of CRCs diagnosed between 2009 and 2011 in London (N = 943), Sheringham et al^12^ found that the odds of an emergency presentation were lower when patients had seen their PCC for any reason. Patients diagnosed through emergency routes presented to primary care later and used urgent care services more often than patients diagnosed through other routes. Individuals without a PCC may be reluctant or unable to seek medical attention until their symptoms are too severe to ignore; the ED becomes their primary point of entry into the health care system.

We found that individuals with right-sided CRC were more likely to present with OPE. The rectum is more capacious and less likely to be associated with obstruction. In addition, the rectum is thicker walled and mostly extraperitoneal and less likely to be associated with perforations.^46^ Rectal cancers are more likely to lead to rectal bleeding, which may lead to earlier diagnostic workup compared with subtle symptoms due to right-sided CRC. In addition, CRC screening is less effective for right-sided CRCs.^47,48,49,50^ Higher rates of OPE among women and older individuals could also be due to a higher proportion of CRC located in the right colon in older individuals and women in general, although this association persisted in site-adjusted analyses. Other reasons are not obvious and merit additional qualitative studies of those with OPE.

### Strengths and Limitations

The results of this study should be interpreted in the context of its strengths and limitations. We used data from previously validated population-based administrative health databases.^25,26,51,52^ Regardless, there may have been misclassification of patients with OPE owing to coding errors. We did not conduct a medical record review to evaluate the accuracy of the *ICD-10* codes used to identify OPE. However, we did use codes that were used in prior studies.^13,14^ This was an observational study and the potential for residual confounding by unmeasured or unrecognized factors exists. Because nonprogram FOBT and ED data were not available for non-Winnipeg residents, the analysis was restricted to Winnipeg. Finally, we used area-level income as a proxy measure for individual-level income which may have attenuated the association between individual income and OPE owing to the misclassification of a few individuals’ actual income. However, prior studies in Manitoba have shown substantial correlation between neighborhood-level income and a self-reported income.^28,29^

## Conclusions

Reducing emergency presentations is an important step in reducing CRC mortality. In Winnipeg, Manitoba, the rate of emergency hospital admissions prior to a CRC diagnosis has decreased. The rate of obstructions and perforations has not decreased. Disparities by income for OPE are present despite organized screening and universal health care. Nevertheless, regular health care contact and a history of CRC screening decreases the likelihood of OPE. These results make a strong argument for targeted screening strategies that focus on lower-income neighborhoods where individuals are at higher risk of OPE.

## Notes

Supplement. **eTable 1.** Factors Associated With an Obstruction, Perforation, or Emergency Hospital Admissions (OPE) or Emergency Department Visits for Individuals Diagnosed With Colorectal Cancer, 52 to 74 Years of Age, Winnipeg, 2007-2015 (n = 1,861) **eTable 2.** Factors Associated With an Obstruction, Perforation, or Emergency Hospital Admissions (OPE) or Emergency Department Visits or Stage IV Diagnosis for Individuals Diagnosed With Colorectal Cancer, 52 to 74 Years of Age, Winnipeg, 2007-2015 (n = 1,861)
