# Supplementary material for: Time Trends in the Diagnosis of Colorectal Cancer With Obstruction, Perforation, and Emergency Admission After the Introduction of Population-Based Organized Screening
Source: JAMA Netw Open. 2020 May 26;3(5):e205741. doi: 10.1001/jamanetworkopen.2020.5741 (PMC7251446; doi:10.1001/jamanetworkopen.2020.5741)
Supplement: Supplement. — eTable 1. Factors Associated With an Obstruction, Perforation, or Emergency Hospital Admissions (OPE) or Emergency Department Visits for Individuals Diagnosed With Colorectal Cancer, 52 to 74 Years of Age, Winnipeg, 2007-2015 (n = 1,861) eTable 2. Factors Associated With an Obstruction, Perforation, or Emergency Hospital Admissions (OPE) or Emergency Department Visits or Stage IV Diagnosis for Individuals Diagnosed With Colorectal Cancer, 52 to 74 Years of Age, Winnipeg, 2007-2015 (n = 1,861) [file jamanetwopen-3-e205741-s001.pdf]

Supplementary Online Content

Decker KM, Lambert P, Nugent Z, Biswanger N, Samadder J, Singh H. Time trends in the diagnosis of colorectal cancer with obstruction, perforation, and emergency admission after the introduction of population-based organized screening. *JAMA Netw Open.* 2020;3(5):e205741. doi:10.1001/jamanetworkopen.2020.5741

eTable 1. Factors Associated With an Obstruction, Perforation, or Emergency Hospital Admissions (OPE) or Emergency Department Visits for Individuals Diagnosed With Colorectal Cancer, 52 to 74 Years of Age, Winnipeg, 2007-2015 (n= 1,861)
eTable 2. Factors Associated With an Obstruction, Perforation, or Emergency Hospital Admissions (OPE) or Emergency Department Visits or Stage IV Diagnosis for Individuals Diagnosed With Colorectal Cancer, 52 to 74 Years of Age, Winnipeg, 2007-2015 (n= 1,861)

This supplementary material has been provided by the authors to give readers additional information about their work.

© 2020 Decker KM et al. *JAMA Network Open.*

eTable 1. Factors Associated With an Obstruction, Perforation, or Emergency Hospital Admissions (OPE) or Emergency Department Visits for Individuals Diagnosed With Colorectal Cancer, 52 to 74 Years of Age, Winnipeg, 2007-2015 (n= 1,861)

Variable		Univariable		Multivariable	
		OR (95% CI)	*P* value[a]	OR (95% CI)	*P* value
Sex	Female	1.28 (1.05-1.57)	0.02	1.24 (0.98-1.54)	0.08
	Male	Ref		Ref	
Age		1.02 (1.00-1.03)	0.06	1.03 (1.01-1.05)	0.01
Income Quintile	IQ1 (lowest)	2.29 (1.65-3.18)	<0.001	1.82 (1.26-2.63)	<0.001
	IQ2	1.94 (1.40-2.70)		1.65 (1.14-2.39)	
	IQ3	1.35 (0.96-1.91)		1.11 (0.75-1.62)	
	IQ4	1.12 (0.87-1.70)		0.98 (0.67-1.43)	
	IQ5 (highest)	Ref		Ref	
Stage	I,II	0.28 (0.22-0.36)	<0.001	0.34 (0.25-0.45)	<0.001
	III	0.33 (0.25-0.42)		0.38 (0.28-0.50)	
	IV	Ref		Ref	
Tumour location	Caecum, ascending colon, hepatic flexure	2.27 (1.73-2.98)	<0.001	3.19 (2.33-4.36)	<0.001
	Transverse colon, splenic flexure	2.87 (1.96-4.20)		4.57 (2.96-7.05)	
	Descending colon, sigmoid colon	1.58 (1.18-2.12)		1.94 (1.39-2.71)	
	Recto-sigmoid junction	1.48 (0.98-2.23)		1.63 (1.03-2.59)	
	Rectum	Ref		Ref	

© 2020 Decker KM et al. *JAMA Network Open.*

Continuity of care[b]	≥50%	0.89 (0.62-1.27)	<0.001	0.98 (0.65-1.49)	0.91
	Fewer than 3 visits	1.55 (1.02-2.36)		1.10 (0.60-2.01)	
	<50%	Ref		Ref	
Screening history	Up-to-date[c]	0.32 (0.26-0.39)	<0.001	0.37 (0.29-0.47)	<0.001
	Not up-to-date	Ref		Ref	
	Program FOBT	0.36 (0.23-0.58)	0.001		
	None	Ref			
	Colonoscopy	0.67 (0.49-0.90)	0.01		
	None	Ref			
	Flexible sigmoidoscopy	0.41 (0.14-1.20)	0.10		
	None	Ref			
	Non-program FOBT	0.38 (0.31-0.48)	<0.001		
	None	Ref			
Co-morbidity	0	2.92 (1.51-5.66)	<0.001	2.33 (1.09-5.00)	0.08
	1	Ref		Ref	
	2	1.23 (0.64-2.35)		1.36 (0.64-2.89)	
	3,4,5	1.33 (0.73-2.45)		1.25 (0.60-2.60)	
Era	2007-2010	1.12 (0.92-1.37)	0.26	0.99 (0.78-1.24)	0.90
	2011-2015	Ref		Ref	
Primary care clinician visits					
31-90 days	1 or more	0.63 (0.51-0.78)	<0.001	0.66 (0.51-0.85)	0.002
	0	Ref		Ref	
91-181 days	1 or more	0.76 (0.61-0.94)	0.01	1.02 (0.77-1.35)	0.88
	0	Ref		Ref	

© 2020 Decker KM et al. *JAMA Network Open.*

182-272 days	1 or more	0.86 (0.70-1.06)	0.16	1.16 (0.86-1.56)	0.33
	0	Ref		Ref	
273-365 days	1 or more	0.90 (0.73-1.11)	0.32	1.25 (0.93-1.67)	0.14
	0	Ref		Ref	
Specialist visits					
31-90 days	1 or more	0.44 (0.35-0.56)	<0.001	0.45 (0.34-0.61)	<0.001
	0	Ref		Ref	
91-181 days	1 or more	0.66 (0.52-0.85)	0.001	0.70 (0.51-0.97)	0.03
	0	Ref		Ref	
182-272 days	1 or more	0.90 (0.69-1.18)	0.45	1.09 (0.77-1.55)	0.64
	0	Ref		Ref	
273-365 days	1 or more	0.97 (0.73-1.27)	0.80	1.38 (0.96-1.98)	0.08
	0	Ref		Ref	
Hospital Admissions					
31-90 days	1 or more	1.56 (0.92-2.66)	0.10	1.75 (0.92-3.35)	0.09
	0	Ref		Ref	
91-181 days	1 or more	1.49 (0.87-2.55)	0.15	1.56 (0.82-2.98)	0.17
	0	Ref		Ref	
182-272 days	1 or more	2.00 (0.98-4.91)	0.06	2.37 (1.03-5.48)	0.04
	0	Ref		Ref	
273-365 days	1 or more	0.92 (0.44-1.93)	0.83	0.56 (0.23-1.40)	0.21
	0	Ref		Ref	

Notes: OR – Odds Ratio; CI – Confidence Interval; FOBT = fecal occult blood test; IQ1=income quintile level 1 (lowest), IQ2=income quintile level 2, IQ3=income quintile level 3, IQ4=income quintile 4, IQ5=income quintile 5 (highest); [a]Type 3 *p* value reported. [b]Continuity of care

© 2020 Decker KM et al. *JAMA Network Open.*

includes primary care clinician visits in the six to 30 months prior to diagnosis. [c]Up-to-date screening is defined as a program or non-program FOBT in the previous 2 years, flexible sigmoidoscopy in the previous 5 years, or a colonoscopy in the previous 10 years (excluding non-program FOBTs, flexible sigmoidoscopies and colonoscopies in the 3 months prior to diagnosis).

© 2020 Decker KM et al. *JAMA Network Open.*

eTable 2. Factors Associated With an Obstruction, Perforation, or Emergency Hospital Admissions (OPE) or Emergency Department Visits or Stage IV Diagnosis for Individuals Diagnosed With Colorectal Cancer, 52 to 74 Years of Age, Winnipeg, 2007-2015 (n= 1,861)

Variable		Univariable		Multivariable	
		OR (95% CI)	*P* value[a]	OR (95% CI)	*P* value
Sex	Female	1.19 (0.98-1.44)	0.07	1.21 (0.98-1.49)	0.08
	Male	Ref		Ref	
Age		1.01 (1.00-1.04)	0.15	1.02 (1.00-1.04)	0.006
Income Quintile	IQ1 (lowest)	2.13 (1.58-2.88)	<0.001	1.80 (1.30-2.51)	<0.001
	IQ2	1.79 (1.32-2.42)		1.59 (1.15-2.21)	
	IQ3	1.19 (0.87-1.62)		1.02 (0.73-1.43)	
	IQ4	1.24 (0.92-1.67)		1.13 (0.82-1.57)	
	IQ5 (highest)	Ref		Ref	
Tumour location	Caecum, ascending colon, hepatic flexure	1.57 (1.20-.94)	0.003	1.99 (1.52-2.62)	<0.001
	Transverse colon, splenic flexure	1.72 (1.20-1.94)		2.32 (1.56-3.44)	
	Descending colon, sigmoid colon	1.21 (0.93-1.57)		1.46 (1.09-1.94)	
	Recto-sigmoid junction	1.37 (0.95-1.98)		1.60 (1.07-2.39)	
	Rectum	Ref		Ref	
Continuity of care[b]	≥50%	0.90 (0.65-1.26)	<0.001	0.98 (0.67-1.42)	0.43
	Fewer than 3 visits	1.78 (1.19-2.64)		1.30 (0.76-2.24)	
	<50%	Ref		Ref	
	Up-to-date[c]	0.31 (0.25-0.37)	<0.001	0.34 (0.27-0.42)	<0.001

© 2020 Decker KM et al. *JAMA Network Open.*

Screening history	Not up-to-date	Ref		Ref	
	Program FOBT	0.36 (0.24-0.54)	<0.001		
	None	Ref			
	Colonoscopy	0.54 (0.41-0.72)	<0.001		
	None	Ref			
	Flexible sigmoidoscopy	0.42 (0.17-1.04)	0.06		
	None	Ref			
	Non-program FOBT	0.41 (034-0.50)	<0.001		
	None	Ref			
Co-morbidity	0	1.97 (1.10-3.53)	<0.001	1.28 (0.67-2.47)	0.28
	1	Ref		Ref	
	2	0.88 (0.51-1.53)		0.92 (0.67-2.47)	
	3,4,5	0.85 (0.51-1.43)		0.79 (0.43-1.45)	
Era	2007-2010	1.11 (0.92-1.33)	0.29	0.97 (0.79-1.19)	0.76
	2011-2015	Ref		Ref	
Primary care clinician visits					
31-90 days	1 or more	0.77 (0.63-0.93)	0.008	0.92 (0.72-1.16)	0.48
	0	Ref		Ref	
91-181 days	1 or more	0.67 (0.55-0.82)	<0.001	0.79 (0.62-1.02)	0.07
	0	Ref		Ref	
182-272 days	1 or more	0.84 (0.69-1.01)	0.07	1.25 (0.96-1.63)	0.09
	0	Ref		Ref	
273-365 days	1 or more	0.82 (0.68-1.00)	0.05	1.12 (0.87-1.45)	0.38
	0	Ref		Ref	
Specialist visits					

© 2020 Decker KM et al. *JAMA Network Open.*

31-90 days	1 or more	0.51 (0.41-0.62)	<0.001	0.57 (0.45-0.73)	<0.001
	0	Ref		Ref	
91-181 days	1 or more	0.58 (0.46-0.73)	<0.001	0.59 (0.45-0.79)	<0.001
	0	Ref		Ref	
182-272 days	1 or more	0.85 (0.66-1.09)	0.20	1.04 (0.76-1.43)	0.81
	0	Ref		Ref	
273-365 days	1 or more	0.89 (0.69-1.15)	0.35	1.28 (0.93-1.78)	0.14
	0	Ref		Ref	
Hospital Admissions					
31-90 days	1 or more	1.54 (0.92-2.59)	0.10	1.63 (0.90-2.96)	0.11
	0	Ref		Ref	
91-181 days	1 or more	1.60 (0.95-2.70)	0.08	2.02 (1.11-3.68)	0.02
	0	Ref		Ref	
182-272 days	1 or more	2.36 (1.14-4.90)	0.02	3.05 (1.33-6.99)	0.01
	0	Ref		Ref	
273-365 days	1 or more	0.94 (0.48-1.84)	0.85	0.63 (0.28-1.39)	0.21
	0	Ref		Ref	

Notes: OR – Odds Ratio; CI – Confidence Interval; FOBT = fecal occult blood test; IQ1=income quintile level 1 (lowest), IQ2=income quintile level 2, IQ3=income quintile level 3, IQ4=income quintile 4, IQ5=income quintile 5 (highest); [a]Type 3 *p* value reported. [b]Continuity of care includes primary care clinician visits in the six to 30 months prior to diagnosis. [c]Up-to-date screening is defined as a program or non-program FOBT in the previous 2 years, flexible sigmoidoscopy in the previous 5 years, or a colonoscopy in the previous 10 years (excluding non-program FOBTs, flexible sigmoidoscopies and colonoscopies in the 3 months prior to diagnosis).

© 2020 Decker KM et al. *JAMA Network Open.*
